# A Phenomenal Depiction of Paranoid Schizophrenia With Auditory Hallucinations: A Case Report

**DOI:** 10.7759/cureus.46092

**Published:** 2023-09-27

**Authors:** Gauri Kakar, Ashok M Mehendale, Kamaldeep Sadh, Sanket S Bakshi, Harsh Bodke, Harshil Krishnani

**Affiliations:** 1 Department of Medicine, Jawaharlal Nehru Medical College, Datta Meghe Institute of Higher Education and Research, Wardha, IND; 2 Department of Community Medicine, Jawaharlal Nehru Medical College, Datta Meghe Institute of Higher Education and Research, Wardha, IND; 3 Department of Psychiatry, Jawaharlal Nehru Medical College, Datta Meghe Institute of Higher Education and Research, Wardha, IND

**Keywords:** aggressive behavior, paranoia, neuro-psychiatric syndrome, delusional disorder, schizophrenia

## Abstract

This case report provides a vivid illustration of a schizophrenic case in a 59-year-old man with auditory hallucinations, illuminating the nature of his symptoms. Auditory hallucinations were prominent, and the patient received voices urging him to perform actions that clearly corresponded to his paranoid thoughts. Through comprehensive research and long-term follow-up, this report reveals the complexity of traumatic schizophrenia, highlighting the importance of early recognition and intervention. One must emphasize a multidisciplinary approach, including psychiatric assessment, pharmacotherapy, and psychotherapy. This case report aims to highlight the critical role of comprehensive individual care in improving the patient’s condition and emphasizes the importance of compassionate healthcare practices.

## Introduction

Schizophrenia or dementia praecox is an exponentially growing pathology worldwide, with a prevalence of one in 300 adults [1]. The Greek words skein (split) and phren (mind) are combined to get the English word schizophrenia. Symptoms include hallucinations, delusions, disorganized communication, sluggish planning, decreased motivation, and dulled emotion [2,3]. It is a debilitating collection of neurological illnesses. The etiology of schizophrenia caters to genetics (microdeletion of chromosome 22q11), environment, alteration of brain chemistry (dysregulation of serotonin, dopamine, and glutamate), and abnormal brain anatomy (atrophy) [4,5]. According to the signs and symptoms, schizophrenia has seven kinds [6]. A characteristic depiction of paranoid schizophrenia usually presents as persistent, frequently paranoid delusions, which in the majority of the cases are auditory or visual in nature, though other modalities can have effects [7,8]. Affect, volition, and speech disturbances, as well as catatonic signs, are not noticeable [9]. It is not just the psychiatrists, instead an efficient plethora of healthcare professionals is essential to treat and manage patients with schizophrenia and schizophrenia-like diseases [3,10].

## Case presentation

A 59-year-old male patient reported to the psychiatric outpatient department with chief complaints of being suspicious and fearful toward his family members and constant disturbances in his sleeping pattern. The mental status examination of the patient as soon as he presented to us has been depicted in Table 1.

**Table 1:** Mental Status Examination

Appearance	Age - 59 years; sex - male; body build - poorly nourished; posture - slouched; eye contact - distorted; attentiveness to the examiner - poor; physical abnormalities - none; emotional facial expressions - exaggerated; alertness - exaggerated
Motor	Retardation - absent; agitation - present; abnormal movements - present; gait - normal; catatonia - absent
Speech	Spontaneous, loud
Affect	Unstable, Inappropriate
Thought content	Depressive cognition present, paranoid ideation present
Thought process	Logic - absent, attention - distorted
Perception	Auditory hallucinations - present
Intellect	Global impressions - below average
Insight	Awareness of Illness - absent

The patient complained that he constantly heard certain voices, telling him that his wife had motives to kill him and that his life was endangered. When asked about this to his wife, she said that he believed that she was trying to harm him and was making plans to kill him. She further said that he would misinterpret her daily routine and feel endangered because of them. The patient was anxious throughout the day. He would check all the locks regularly, showed hesitation about eating homemade food made by his wife, and would be suspicious when his wife would go out and meet people. Upon seeing the wife’s side of the family, he would run away. The patient would often question his wife if she spoke to another man, constantly humiliating her, and often questioning her way of dressing. The patient would ask his daughter to keep the wife away from him and get irritable and agitated upon seeing her. When enquired about the case, the wife stated that after their marriage, her mother-in-law would keep telling the patient ill things about his wife. Such episodes would occur if the patient underwent a stressful situation. The patient had been previously referred to a hospital regarding his complaints and was diagnosed as a case of paranoid schizophrenia.

**Figure 1:**
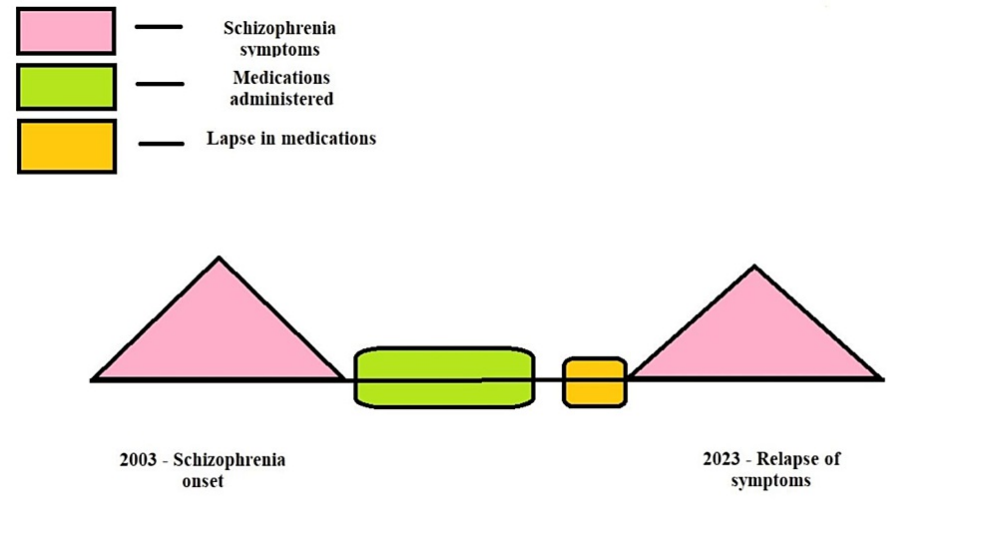
Depiction of the Course of Schizophrenia in This Case The author self-created the image.

According to his family, his illness began 20 years ago, in 2003. The patient underwent treatment, which consisted of a combination therapy of a single dose of two oral antipsychotics per day, and the patient was then shifted to a combination of a long-acting injectable (LAI) antipsychotic and an oral dose of antipsychotic and showed signs of remission. These episodes would enhance and were initiated due to potential stress situations and non-compliance with medication causing mild irritability and persistent suspiciousness. Decreased sleep aggravated his irritability, which worsened his symptoms. Another episode of abnormal behavior was observed when the patient removed his clothes and ran away without a reason. He had to be brought back after tying his hands to a nearby hospital. The patient had to undergo hospital admission for 25 days. The initial episodes that took place earlier continued for a month. With the onset of effective treatment and patient compliance, he was finally symptom-free until three days back, when his symptoms drastically aggravated. The course of the prognosis is shown in Figure 1. The patient also had jumped from the roof of his house with the fear that his wife was trying to kill him, due to which he had a bleeding injury on his left foot.

**Figure 2:**
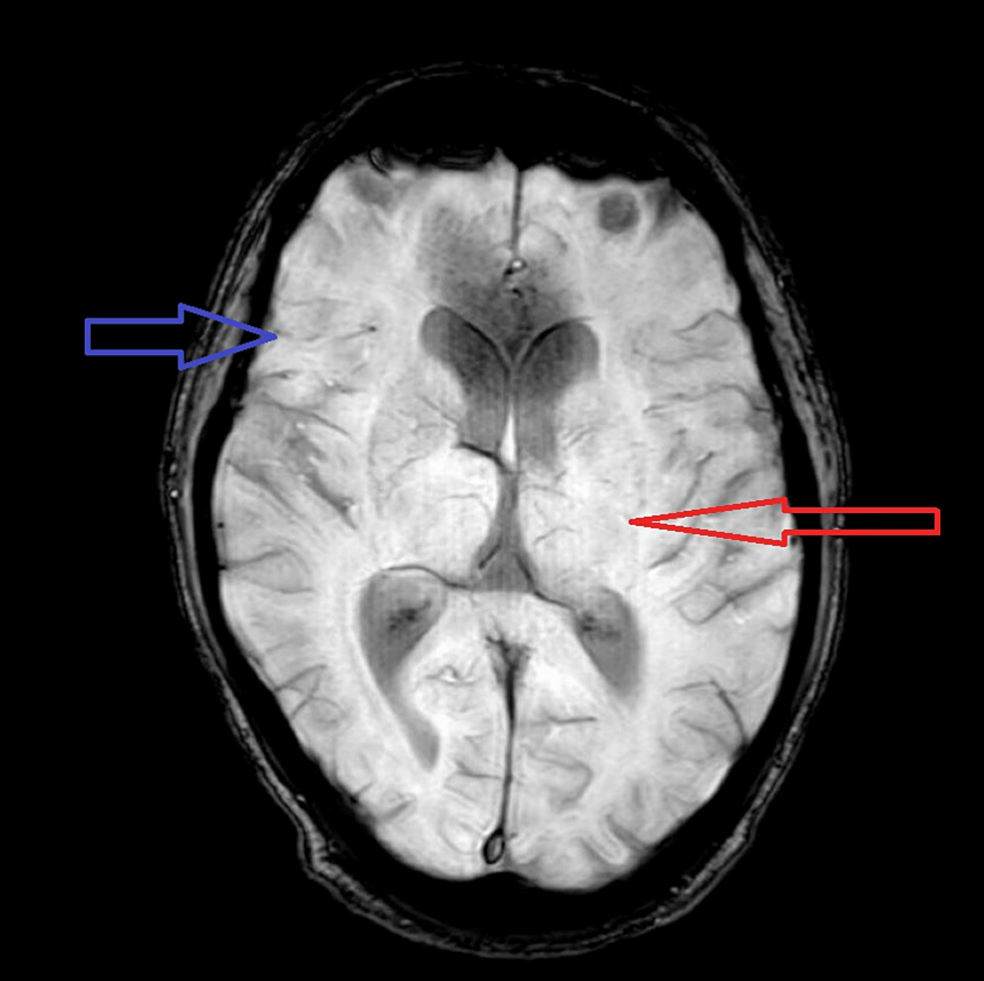
MRI Scan Showing Age-Related Atrophic Changes and Periventricular Hyperintensity MRI, magnetic resonance imaging

The patient’s blood workup conducted was within normal limits. A magnetic resonance imaging (MRI) scan was done. The MRI revealed the prominence of sulcogyral spaces. T2 flair hyperintensities were evident in the periventricular region and bilateral centrum semiovale. Along with this, a computed tomography (CT) scan was done. The CT scan revealed the prominence of bilateral lateral ventricles and focal white matter hypodensity involving the subcortical left frontal lobe. The patient was further evaluated concerning the ICD-10 screening questionnaire, which assisted in confirming the previous diagnosis of paranoid schizophrenia. The impression of age-related atrophic changes with small vessel ischemic disease is shown in Figure 2. 

When his actions owed to violence, he was administered the treatment regimen of intra-muscular (IM) haloperidol 10 mg and intravenous (IV) diazepam 10 mg for quick tranquilization. After 24 hours, the same amount of dosage in the IM form of haloperidol 10 mg and IM form of diazepam 10 mg was administered. Both these drug administrations had close to no positive outcomes. The next step was to switch his medication to IM midazolam 7.5 mg and IM haloperidol 5 mg. Along with clonazepam 0.5 mg pills to treat his sleep problems, the patient was also given olanzapine 15 mg tablets for the treatment of schizophrenia on the day of admission. Nurses reported that the patient refused to take his olanzapine 15 mg tablet for a few days because he was restless, even though the patient's meds were given under directly observed treatment (DOT). 

## Discussion

Aggression and schizophrenia have a long history of presenting together, which has implications for public policy, research, and therapy [11]. Effectively controlling and reducing aggressiveness benefit patients as well as their families and create a less dangerous environment. There were a few characteristic presenting features in our patient that are noteworthy. Due to non-compliance, he experienced a recurrence of his schizophrenia and had symptoms of active psychopathology. Clinically, we were able to prove that the patient had active paranoid symptoms, which were brought on by his deviant belief and then systematized by voices telling him that he had a threat to his life and that his wife was trying to kill him. Hallucinations, in particular commanding auditory hallucinations, enhance the likelihood of his aggressive behavior because the patient is more likely to obey an order when it comes from a familiar voice [12]. Patients with persecution delusions may also proactively assault the victim in self-defense if they believe someone is planning to injure or attack them [13]. The MRI of the patient depicted peculiar changes in the brain anatomy, showing periventricular hyperintensities, which are a measure of microstructural changes in the neurotopography, suggesting progressive demyelination. 

Paranoid schizophrenia's "control override" or passivity phenomenon causes schizophrenic patients to feel that someone is in charge of their ideas or minds, which intensifies violent conduct [14]. This action supports past research that suggested aggressiveness, particularly delusions and hallucinations, occur in reaction to psychotic experiences and that the content of the psychotic symptom may be crucial in connection to risky behavior [5,11]. In a previous study conducted, it was also shown that the kind and severity of a patient's psychopathology are significant determinants influencing aggressiveness [15]. 

## Conclusions

Increased risk for violent offenses in paranoid schizophrenia is due to both current symptoms and related social issues. Therefore, such problems should be the focus of addressing this group of patients. To do this, the service delivery system must be reorganized, and healthcare staff must get new training. Further research and clinical experience are needed to tailor treatment strategies to improve the overall quality of life of individuals with this complex mental illness. 
